# T-cell immunodeficiency and reconstruction based on TCR rearrangement analysis in hematological malignancy: update from 2011 ASH annual meeting

**DOI:** 10.1186/1756-8722-5-S1-A3

**Published:** 2012-04-25

**Authors:** Yangqiu Li

**Affiliations:** 1 Institute of Hematology, Medical College, and Key Laboratory for Regenerative Medicine of Ministry of Education, Jinan University, Guangzhou 510632, China

## Introduction

Poor cellular immune function may relate to carcinogenic processes and to worse prognosis in solid tumor patients as well as in leukemia. Moreover, the progression of tumor might further induce the cellular immune suppression. Therefore, a set of molecular immunological techniques to analyze and monitor the changes of host T-cell immune status is needed, which can fully characterize the feature of T-cell immunodeficiency in different malignancies, providing information and direction for immune reconstruction, in particular for enhancement the specific anti-tumor immune function.

## The feature of T-cell immunodeficiency in hematological malignancies

In recent years, molecular analysis of the T cell receptor (TCR) utilization feature based on the principle of TCR α, β, γ and δ gene rearrangement and deletion rearrangement, has proven to be an effective technique for studying the distribution of T cell repertoire, the diversity of TCR subfamilies [1,2], the antigen specific expansion of T-cell clones and the recent thymic output function [3,4]. This in turn can help to characterize the feature of host T cell immune status, the identification of T-cell populations of interest in cancer, as well as the peripheral immune repertoire reconstitution after hematopoietic stem cell transplantation (HSCT).

T-cell immunodeficiency is a common feature in different hematological malignancies, including the absence of TCR Vα and Vβ subfamilies, decreased diversity of TCR repertoires, reduced thymic recent output function (naïve T cells) and lower frequencies of TCR subfamily naïve T cells. An impaired thymic export function and, as a consequence, altered ability to maintain T cell homeostasis may play an important pathogenic role in hematological malignancies. On the other hand, clonally expanded T cells could be identified in some TCR subfamilies in leukemia patients, which display specific anti-leukemia cytotoxicity like WT1 or BCR-ABL specific CTL, indicating that specific anti-leukemic T cells could be generated in vivo. This suggests that the host could have the ability of specific immune response to leukemia associated antigens, despite of T cell immunodeficiency.

## T-cell immune reconstitution and establishment of specific anti-tumor and virus immunity

Prolonged period of immunodeficiency and poor immune reconstitution after stem cell transplantation place patients at high risk for viral infection and disease relapse, resulting in significant morbidity and mortality. Reversion of the cellular immunodeficiency is one of the crucial steps for improvement the outcome of tumor therapy in hematological malignancies. Moreover, the T-cell immune reconstitution is a key determinacy of long-term outcome in patients with hematological malignancies post chemotherapy or stem cell transplantation.

T-cell immune reconstitution requests not only the recovery of the comprehensive T-cell immunity, a broad TCR repertoire and recent thymic emigrants, more importantly, also the enhancement of the specific anti-tumor cellular immune function, which plays determinant role on elimination of minimal residual disease, relapse prevention and improvement of prognosis in hematological malignancies. Antigen specific T-cell immune reconstitution could be carried out by active (cancer vaccine) or adoptive immunotherapy (T-cells transfusion) [5-8]. Cancer vaccines induce expansion and functional differentiation of tumor antigen-specific effectors and memory cells. The latter are particularly relevant for prevention of disease relapse. Adoptive antigen specific immunotherapy is one of the best approaches for tumor immunotherapy. The antigen specific CTL can directly kill tumor cells, ignoring the host immune status.

Antigen specific CTL could be amplified by cellular or gene engineering techniques. Peptide -specific stimulation in vitro can induce high-affinity CTL (auto- or allogenetic) capable of recognizing tumor cells expressing the appropriate tumor antigen. For example, Epstein-Barr virus (EBV)-specific CTL were used to treat the post transplantation lymphoproliferative disease (PTLD) or EBV+ lymphoma, CMV-specific CTL were used to establish anti-CMV immunity in immunodeficiency patients post allogeneic stem cell transplantation [9,10]. Genetically-modified CTL were obtained by engineering antigen specific TCR gene, thus altering their original antigen specificity and arming them with new cytotoxicity for tumor cells. The approach provides a new strategy for adoptive specific immunotherapy in malignancies and so on. A lot of TCR-modified CTL against different leukemia and lymphoma were developed, like mHagHA-2, EBV, WT1, CML or DLBCL-specific TCR modified CTL [5-8,11,12], as well as the single-chain antibody-derived chimeric antigen receptors (CARs) modified T cells that specifically recognize surface molecules expressed on malignant B cells (CD19) or acute myeloid leukemia cells (CD33) independent from HLA [13,14].

In summary, dynamic detection of the alteration of host immune function in patients is important for the defense of host against neoplastic transformation and so on. The new techniques of tumor immunology, molecular biology and increased knowledge of the optimal methodology for generation of T-cell products and optimization of gene therapy approaches make it possible to enhance the function of adoptively transferred T cells. This enhances tumor- specific response and can reverse the host immunodeficiency status.
